# Identification of potential drug targets for vascular dementia and carotid plaques by analyzing underlying molecular signatures shared by them

**DOI:** 10.3389/fnagi.2022.967146

**Published:** 2022-10-03

**Authors:** Jun Shu, Yiqing Ren, Wen Tan, Wenshi Wei, Li Zhang, Jie Chang

**Affiliations:** ^1^Department of Neurology, Cognitive Disorders Center, Huadong Hospital Affiliated to Fudan University, Shanghai, China; ^2^Department of Endocrinology, Huadong Hospital Affiliated to Fudan University, Shanghai, China

**Keywords:** vascular dementia, carotid plaques, immune cell infiltration, gene-drug interaction analysis, bioinformatics

## Abstract

**Background:**

Vascular dementia (VaD) and carotid atherosclerotic plaques are common in the elderly population, conferring a heavy burden on families and society. Accumulating evidence indicates carotid atherosclerotic plaques to be a risk factor for VaD. However, the underlying mechanisms for this association are mainly unknown.

**Materials and methods:**

We analyzed temporal cortex gene expression data of the GSE122063 dataset and gene expression data of the GSE163154 dataset to identify commonly differentially expressed genes (DEGs). Then we performed functional enrichment analysis, immune cell infiltration and evaluation, correlation analysis between differentially expressed immune-related genes (DEIRGs) and immune cells, receiver operating characteristic (ROC) analysis, and drug-gene analysis.

**Results:**

We identified 41 overlapped DEGs between the VaD and carotid atherosclerosis plaque datasets. Functional enrichment analyses revealed that these overlapped DEGs were mainly enriched in inflammatory and immune-related processes. Immunocyte infiltration and evaluation results showed that M0 macrophages, M2 macrophages, and T cells gamma delta had a dominant abundance in carotid atherosclerosis plaque samples, and M0 macrophages showed a significantly different infiltration percentage between the early and advanced stage plaques group. Resting CD4 memory T cells, M2 macrophages, and naive B cells were the top three highest infiltrating fractions in VaD. Furthermore, B cells and NK cells showed a different infiltration percentage between VaD and matched controls. We identified 12 DEIRGs, and the result of correlation analysis revealed that these DEIRGs were closely related to differentially expressed immune cells. We identified five key DEIRGs based on ROC analysis. The drug-gene interaction analysis showed that four drugs (avacopan, CCX354, BMS-817399, and ASK-8007) could be potential drugs for VaD and carotid atherosclerotic plaques treatment.

**Conclusion:**

Collectively, these findings indicated that inflammatory and immune-related processes be a crucial common pathophysiological mechanism shared by VaD and carotid plaques. This study might provide new insights into common molecular mechanisms between VaD and carotid plaques and potential targets for the treatment.

## Introduction

Vascular dementia (VaD) is not a specific nosological entity but a syndrome encompassing a number of diseases caused by insufficient blood supply to the brain. It is widely regarded as the second most common type of dementia after Alzheimer’s disease (AD), and there are no licensed treatments for it (Iadecola et al., 2019). Carotid atherosclerotic plaques is a chronic inflammatory disease and might be implicated in VaD risk. A recent study found that subclinical carotid plaque burden is related to reduced brain metabolism (Cortes-Canteli et al., 2021). A meta-analysis of three studies assessing carotid plaque showed an association between carotid plaque and impaired cognitive function (Anbar et al., 2022). However, little is known about the underlying mechanisms responsible for this association.

Previous studies showed that VaD and carotid plaque shared some common risk factors, including diabetes, cholesterol levels, smoking, and a sedentary lifestyle (Baumgart et al., 2015). Bioinformatic analysis of transcriptomes has been instrumental in investigating shared biological mechanisms of comorbid diseases. Chung and Lee (2021) analyzed blood transcriptomic signatures between AD and diabetes mellitus (DM) and revealed common dysregulated gene modules between AD and DM. Several studies conducted transcriptome analysis on VaD and found that immunity and inflammation, oxidative stress, and apoptosis were involved in VaD (Chen et al., 2022; Shu et al., 2022; Wang et al., 2022). Recent studies also found that immune and inflammation process played vital roles in the progression (Dong et al., 2017), instability (Zheng et al., 2022), and rupture (Li et al., 2022) of carotid atherosclerotic plaques based on bioinformatic analysis. However, to our knowledge, few studies have compared transcriptome signatures between VaD and carotid atherosclerotic plaques. Therefore, our study aimed to analyze transcriptome signatures between VaD and carotid atherosclerotic plaques to shed light on the underlying pathology of cognitive decline associated with carotid atherosclerotic plaques and provide new candidate targets for therapeutic intervention.

We analyzed the two datasets GSE122063 and GSE163154 to identify commonly differentially expressed genes (DEGs). Then we performed functional enrichment analysis, immunocyte infiltration and evaluation, receiver operating characteristic (ROC) analysis, and drug-gene analysis to identify underlying common pathophysiological mechanisms and potential targets for the treatment.

## Materials and methods

### Data acquisition and descriptions

The gene expression profiles of GSE122063, GSE163154, and GSE28829 were downloaded from the Gene Expression Omnibus (GEO)^1^ (Barrett et al., 2013). The dataset GSE122063 contains gene expression profiles of the frontal cortex and temporal cortex samples from 18 VaD and 22 matched controls. The GSE163154 dataset includes 16 no-intraplaque hemorrhage (the early-stage) samples and 27 intraplaque hemorrhage (the advanced-stage) samples from symptomatic patients undergoing carotid endarterectomy surgery. The GSE28829 dataset consists of 16 clinically proven advanced-stage atheromatous carotid plaques and 13 early-stage atheromatous carotid plaques. These three datasets were selected because they all contained both case groups and matched control groups, and the number of case samples and control group samples were both greater than 10, and the update time of these datasets was after 2019. In the current study, the gene expression data of the temporal cortex from VaD and matched control patients in the GSE122063 dataset and the GSE163154 dataset were designated as the exploring datasets. The gene expression data of the frontal cortex in the GSE122063 and the GSE28829 dataset were used as the validating datasets.

### Identification of differentially expressed genes

Differentially expressed genes (DEG) of the microarray dataset GSE122063 and GSE163154 were identified with NCBI’s GEO2R tool^2^ using the Limma package. A Venn diagram (Bardou et al., 2014) was used to identify the overlapping DEGs between the GSE122063 dataset and the GSE163154 dataset.

### Gene ontology and pathway enrichment analysis

Gene Ontology (GO) and pathway enrichment analysis were performed to obtain a better understanding of the biological mechanisms of the overlapping genes using Metascape^3^ (Zhou et al., 2019). The Kyoto Encyclopedia of Genes and Genomes (KEGG) (Kanehisa et al., 2017), Reactome (Jassal et al., 2020), and WikiPathways (Martens et al., 2021) databases were used for pathway annotations.

### Immune cell infiltration and evaluation

The relative proportions of 22 infiltrating immune cell types were estimated using the CIBERSORT algorithm, by which the normalized gene expression matrix can be transformed into the composition of infiltrating immune cells (Chen et al., 2018). LM22 was used as a reference expression signature with 1,000 permutations. The correlation between immune cells was determined, and the R package ggplot2 visualized the results in R software. Significant immune cells were screened with the threshold Wilcoxon test at the *p*-value < 0.05.

### Correlation analysis between differentially expressed immune-related genes and immune cells

A total of 1,793 immune-related genes (IRGs) were downloaded from ImmPort (Bhattacharya et al., 2018). The overlapping DEGs were intersected with IRGs to obtain differentially expressed immune-related genes (DEIRGs). The relationship between DEIRGs expression and relative percentages of immune cells was investigated using the Spearman method. A *p*-value < 0.05 was considered statistically significant.

### Receiver operating characteristic analysis

To identify the potential key DEIRGs, we evaluated the diagnostic values of these DEIRGs in VaD and carotid plaques by applying “pROC” packages. The key DEIRGs were defined as the area under the curve (AUC) higher than 0.8 in both GSE122063 and GSE163154 datasets. Frontal cortex expression data of the GSE122063 and the GSE28829 dataset were used as the validating datasets.

### Gene-drug interaction analysis

The key DEIRGs were imported into the Drug-Gene Interaction Database (DGIdb)^4^ (Freshour et al., 2021) as potential targets to search for existing drugs that could interact with them. Drugs that showed specific types of interactions with the key genes were selected. To identify related clinical trials, the selected drugs were input into the ClinicalTrials.govregistry (clinicaltrials.gov) which contains over 329,000 trials worldwide.

## Results

### Identification of differentially expressed genes

**FIGURE 1:**
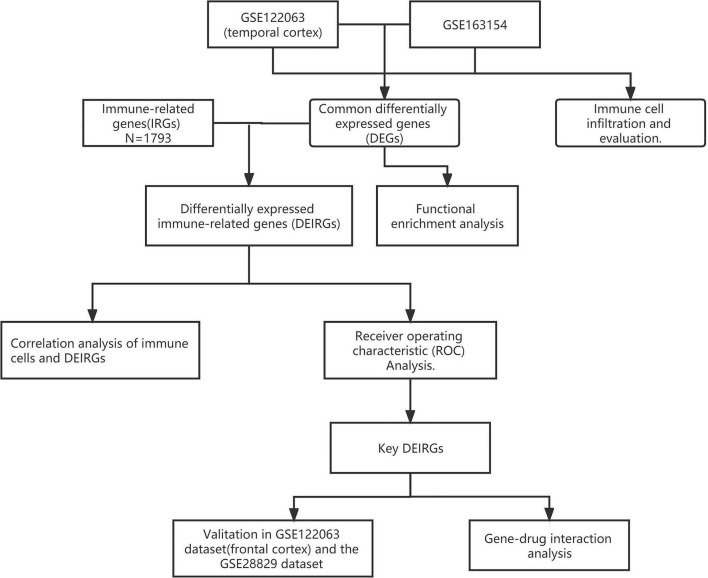
Flowchart of the analytical steps of this study.

A schematic representation of this study is shown in Figure 1.

**FIGURE 2:**
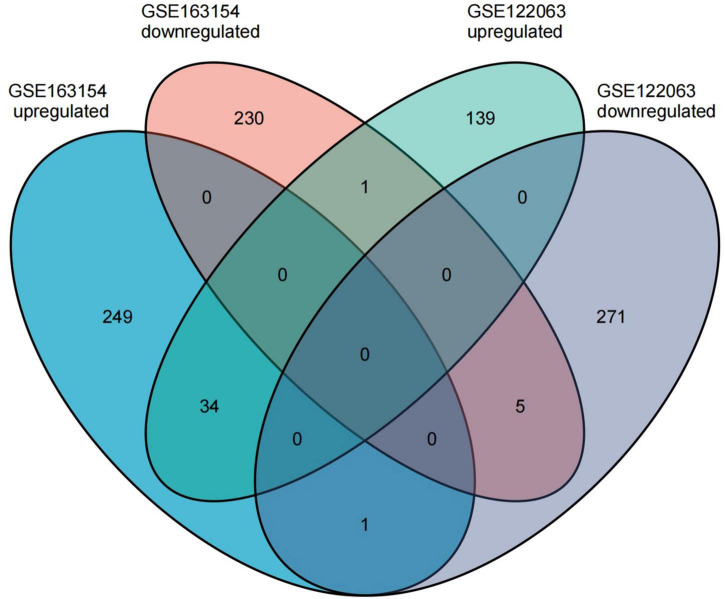
The common differentially expressed genes (DEGs) were identified between the two datasets.

The GSE163154 dataset and temporal cortex gene expression data of the GSE122063 dataset were analyzed to identify DEGs, respectively. The GSE163154 dataset detected 284 upregulated and 236 downregulated genes, and temporal cortex gene expression data of the GSE122063 dataset detected 174 upregulated and 277 downregulated genes. A total of 41 overlapping DEGs were identified between the above two datasets. Among them, 34 genes were upregulated and five were downregulated in both datasets; one gene was upregulated in the GSE163154 dataset but downregulated in the GSE122063 dataset, and one gene was downregulated in the GSE163154 dataset but upregulated in the GSE122063 dataset (Figure 2 and Table 1).

**TABLE 1:** Overlapping differentially expressed genes both in vascular dementia (VaD) and carotid atherosclerotic plaques datasets.

DEGs (the number of genes)	Gene symbols
GSE163154 upregulated and GSE122063 upregulated (34)	HMOX1, IFI30, CD163, RGS1, LCP1, C5AR1, CD68, CD14, CD300A, CFD, CPVL, LILRB3, C1QC, CCR1, SPP1, FPR3, C1QA, STAB1, SLAMF8, ITGB2, RNASET2, C1QB, FYB, MS4A6A, SLC16A3, CYBB, IL10RA, EVI2B, VSIG4, LY96, PARVG, RHBDF2, CCL2, and HSPA6
GSE163154 upregulated and GSE122063 downregulated (1)	CD33
GSE163154 downregulated and GSE122063 upregulated (1)	AQP1
GSE163154 downregulated and GSE122063 downregulated (5)	FIBIN, AIF1L, FOXC1, CARTPT, and ACTG2

### Gene ontology and pathway enrichment analysis

**FIGURE 3:**
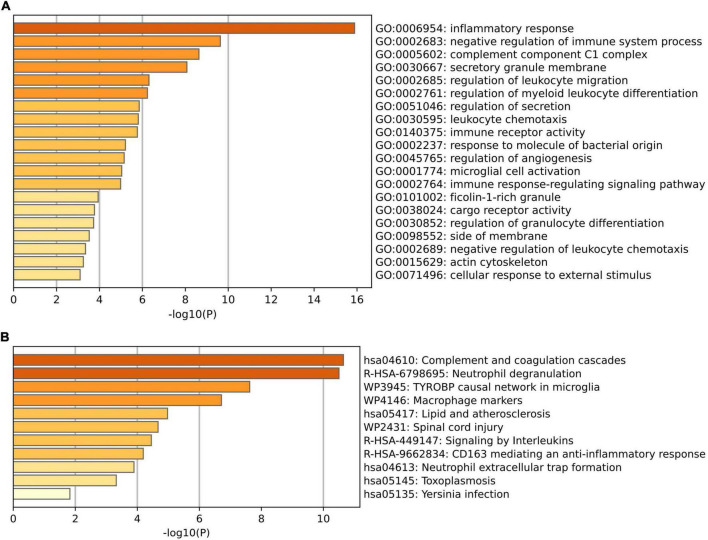
Functional enrichment analysis of the overlapped differentially expressed genes (DEGs) between VaD and carotid atherosclerotic plaques datasets. **(A)** Significant enriched GO terms of the overlapped DEGs. **(B)** Significantly enriched pathways of the overlapped DEGs.

To further explore the potential functions of these overlapping genes, functional enrichment analysis was performed using the online database Metascape. The results revealed that these overlapping genes were enriched in inflammatory and immune-related processes, such as inflammatory response, negative regulation of immune system process, regulation of leukocyte migration, regulation of myeloid leukocyte differentiation, and leukocyte chemotaxis (Figure 3A and Table 2). Pathway enrichment analysis revealed that these overlapping genes were mainly involved in complement and coagulation cascades, lipid and atherosclerosis, neutrophil extracellular trap formation, and neutrophil degranulation (Figure 3B and Table 3).

**TABLE 2:** Significantly enriched GO terms of the overlapping differentially expressed genes (DEGs) between vascular dementia (VaD) and carotid atherosclerotic plaques datasets.

Category	Term	Description	*P*-value	Symbols
GO biological processes	GO:0006954	Inflammatory response	0.0000	C1QA, C5AR1, CD14, CD68, CCR1, CYBB, FPR3, HMOX1, ITGB2, CCL2, SPP1, CD163, STAB1, LY96, SLAMF8, AQP1, CFD, IL10RA, and RGS1
GO biological processes	GO:0002683	Negative regulation of immune system process	0.0000	C1QC, CD68, HMOX1, CCL2, CARTPT, LILRB3, CD300A, VSIG4, SLAMF8, RHBDF2, and SPP1
GO biological processes	GO:0002685	Regulation of leukocyte migration	0.0000	C5AR1,CCR1,HMOX1, CCL2,CD300A,SLAMF8, VSIG4,RHBDF2, ITGB2,CD33, AQP1,IFI30,CFD,LCP1
GO biological processes	GO:0002761	Regulation of myeloid leukocyte differentiation	0.0000	C1QC, CCR1, EVI2B, CARTPT, LILRB3, SLAMF8, and SPP1
GO biological processes	GO:0051046	Regulation of secretion	0.0000	AQP1, CD33, HMOX1, ITGB2, SPP1, CARTPT, CD300A, RHBDF2, and LCP1
GO biological processes	GO:0030595	Leukocyte chemotaxis	0.0000	C5AR1, CCR1, ITGB2, CCL2, SLAMF8, FPR3, FOXC1, IL10RA, LILRB3, CARTPT, CD300A, CD33, and CYBB
GO biological processes	GO:0002237	Response to molecule of bacterial origin	0.0000	C5AR1, CD14, CD68, IL10RA, CCL2, LY96, AQP1, SPP1, CD33, CYBB, HMOX1, FYB1, and CD300A
GO biological processes	GO:0045765	Regulation of angiogenesis	0.0000	AQP1, C5AR1, CYBB, FOXC1, HMOX1, STAB1, CD14, FIBIN, SPP1, CD68, CARTPT, RNASET2, and LCP1
GO biological processes	GO:0001774	Microglial cell activation	0.0000	C1QA, C5AR1, ITGB2, LCP1, SLAMF8, HMOX1, CCL2, CD14, and CD68
GO biological processes	GO:0002764	Immune response-regulating signaling pathway	0.0000	C5AR1, CD14, CD33, FPR3, FYB1, LY96, and CCR1
GO biological processes	GO:0030852	Regulation of granulocyte differentiation	0.0002	C1QC, EVI2B, and CCR1
GO biological processes	GO:0002689	Negative regulation of leukocyte chemotaxis	0.0004	CCL2, SLAMF8, C5AR1, AQP1, STAB1, VSIG4, RHBDF2, LY96, CARTPT, and CD300A
GO biological processes	GO:0071496	Cellular response to external stimulus	0.0008	AQP1, CD68, HMOX1, CARTPT, CCL2, FOXC1, and C5AR1
GO cellular components	GO:0005602	Complement component C1 complex	0.0000	C1QA, C1QB, C1QC, C5AR1, CFD, FPR3, FYB1, VSIG4, ITGB2, CCL2, LCP1, ACTG2, HSPA6, SLC16A3, and LILRB3
GO cellular components	GO:0030667	Secretory granule membrane	0.0000	C5AR1, CD14, CD33, CD68, CYBB, ITGB2, LILRB3, CD300A, RGS1, and CCL2
GO cellular components	GO:0101002	Ficolin-1-rich granule	0.0001	CFD, HSPA6, ITGB2, CD300A, CD14, HMOX1, and RNASET2
GO cellular components	GO:0098552	Side of membrane	0.0003	CD14, CD33, CCR1, ITGB2, RGS1, CD163, IL10RA, STAB1, HMOX1, CCL2, CD300A, and LY96
GO cellular components	GO:0015629	Actin cytoskeleton	0.0006	ACTG2, FYB1, LCP1, PARVG, AIF1L, ITGB2, HMOX1, AQP1, and FOXC1
GO molecular functions	GO:0140375	Immune receptor activity	0.0000	C5AR1, CCR1, FPR3, IL10RA, LILRB3, AQP1, HMOX1, CCL2, CARTPT, and SLAMF8
GO molecular functions	GO:0038024	Cargo receptor activity	0.0002	ITGB2, CD163, STAB1, CYBB, IFI30, and HMOX1

**TABLE 3:** Significantly enriched pathways of the overlapping differentially expressed genes (DEGs) between vascular dementia (VaD) and carotid atherosclerotic plaques datasets.

Category	Term	Description	*P*-value	Symbols
KEGG Pathway	hsa04610	Complement and coagulation cascades	0.0000	C1QA, C1QB, C1QC, C5AR1, CFD, ITGB2, VSIG4, FPR3, CD14, LY96, CYBB, CCL2, and HSPA6
KEGG Pathway	hsa05417	Lipid and atherosclerosis	0.0000	CD14, CYBB, HSPA6, CCL2, LY96, HMOX1, SPP1, and IL10RA
KEGG Pathway	hsa04613	Neutrophil extracellular trap formation	0.0001	C5AR1, CYBB, FPR3, ITGB2, HMOX1, CCL2, CD14, ACTG2, and SLC16A3
KEGG Pathway	hsa05145	Toxoplasmosis	0.0005	HSPA6, IL10RA, LY96, and IFI30
KEGG Pathway	hsa05135	Yersinia infection	0.0149	FYB1, CCL2, and ITGB2
Reactome Gene Sets	R-HSA-6798695	Neutrophil degranulation	0.0000	C5AR1, CD14, CD33, CD68, CYBB, CFD, HSPA6, ITGB2, RNASET2, LILRB3, CD300A, FYB1, IFI30, and LY96
Reactome Gene Sets	R-HSA-449147	Signaling by Interleukins	0.0000	CCR1, HMOX1, IL10RA, ITGB2, LCP1, CCL2, IFI30, C5AR1, FPR3, and RGS1
Reactome Gene Sets	R-HSA-9662834	CD163 mediating an anti-inflammatory response	0.0001	CD163, RHBDF2, STAB1, and HMOX1
WikiPathways	WP3945	TYROBP causal network in microglia	0.0000	C1QC, IL10RA, ITGB2, RGS1, SPP1, C5AR1, CFD, VSIG4, and PARVG
WikiPathways	WP4146	Macrophage markers	0.0000	CD14, CD68, CD163, ITGB2, CCL2, LY96, HSPA6, SPP1, CYBB, IL10RA, and CD33
WikiPathways	WP2431	Spinal cord injury	0.0000	AQP1, C1QB, CCL2, and LILRB3

### Immune cell infiltration and evaluation

**FIGURE 4:**
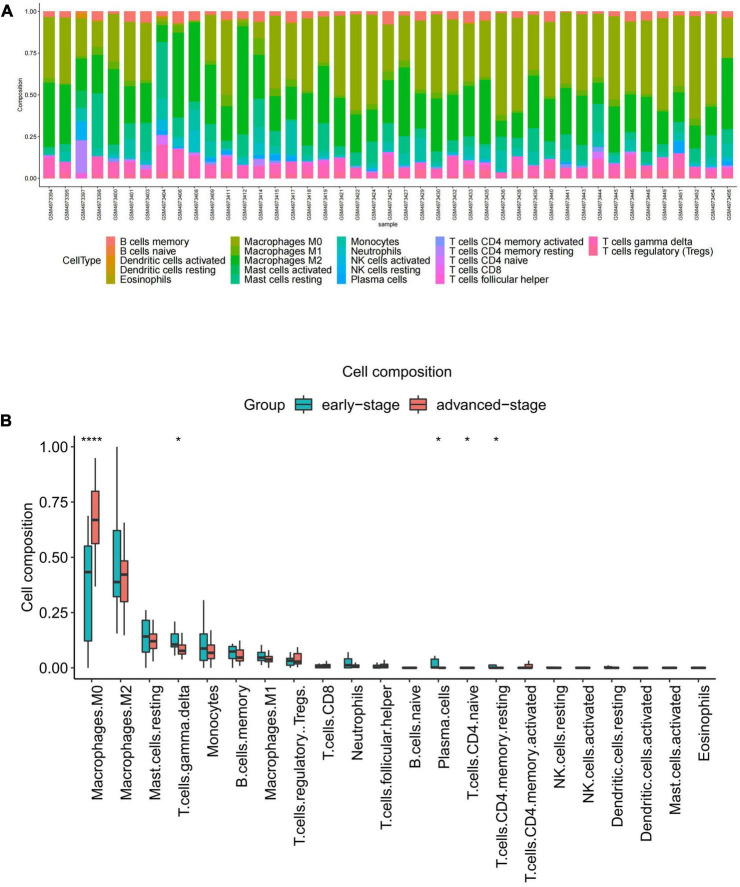
Immune cell Infiltration and evaluation in carotid atherosclerotic plaque samples. **(A)** Relative proportion of immune infiltration of 22 immune cell types in carotid atherosclerotic plaques samples. **(B)** The differential expression of 22 immune cells in the early-stage and advanced-stage carotid plaque samples. Significance level was denoted by *p-value < 0.05, **p-value < 0.01, ***p-value < 0.001.

**FIGURE 5:**
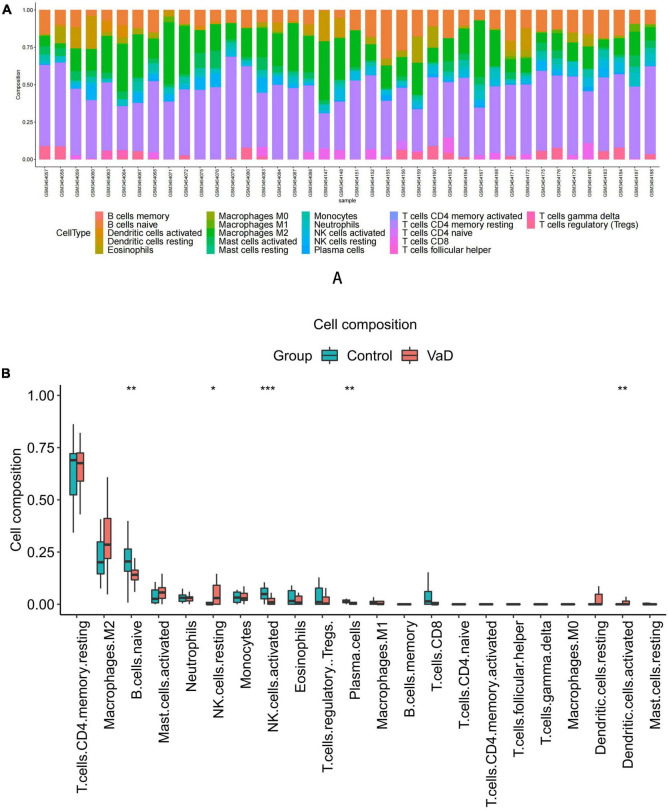
Immune cell Infiltration and evaluation in vascular dementia (VaD) dataset. **(A)** Relative proportion of immune infiltration of 22 immune cell types in VaD and matched controls samples. **(B)** The differential expression of 22 immune cells in the VaD and matched controls samples. Significance level was denoted by *p-value < 0.05, **p-value < 0.01, ***p-value < 0.001.

Functional enrichment analysis indicated that inflammatory and immune processes played a vital role in the development of VaD and carotid atherosclerosis plaques. Therefore, we evaluated the infiltration of 22 immune cell types in the GSE163154 dataset and GSE122063 dataset using the “CIBERSORT” package in R software. Figure 4A showed the relative proportions of 22 immune cells types in 16 early-stage and 27 advanced-stage carotid atherosclerotic plaque samples. The top five highest infiltrating fractions in carotid plaque were M0 macrophages, M2 macrophages, resting mast cells, T cells gamma delta, and monocyte cells. Five immune cells, including M0 macrophages, T cells gamma delta, plasma cells, naive CD4 T cells, and resting CD4 memory T cells, were detected differentially infiltrated between the early-stage and advanced-stage groups (Figure 4B). Figure 5A showed the relative proportions of 22 immune cells types in temporal cortex samples from 18 VaD and 22 matched controls. The top five abundant subtypes were resting CD4 memory T cells, M2 macrophages, naive B cells, activated mast cells, and neutrophils. The percentage of B cells naive, resting NK cells, and activated dendritic cells were significantly higher in the VaD group than in the matched control group, while activated NK cells and plasma cells displayed the opposite proportion (Figure 5B).

**FIGURE 6:**
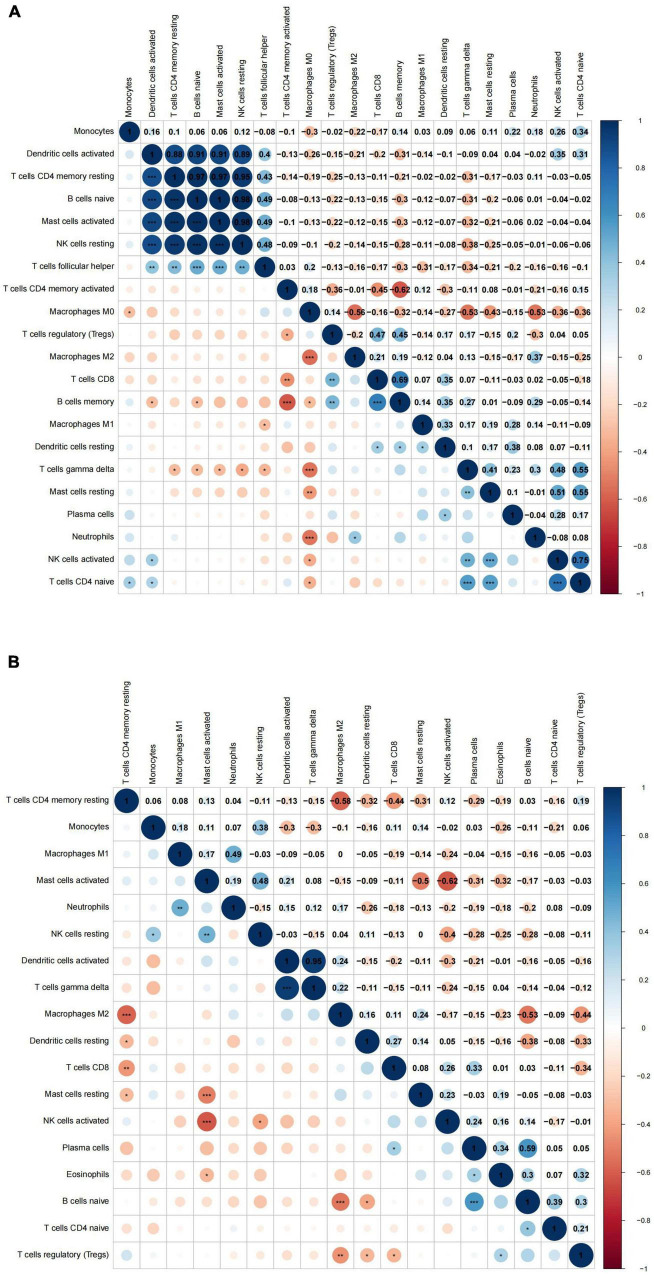
The correlation analysis between 22 immune cell types in the carotid atherosclerotic plaques and vascular dementia (VaD) datasets. **(A)** Heat map of the correlation between 22 immune cell types in the carotid atherosclerotic plaques dataset. **(B)** Heat map of the correlation between 22 immune cell types in the vascular dementia dataset. Significance level was denoted by *p-value < 0.05, **p-value < 0.01, ***p-value < 0.001.

Additionally, we conducted a correlation analysis of 22 immune cells types in VaD dataset and carotid atherosclerosis dataset, and positive and negative correlations between immune cells were displayed in Figure 6. These results revealed that different kinds of immune cells interfered with each other in VaD and carotid atherosclerosis plaques.

### Correlation analysis between differentially expressed immune-related genes and immune cells

**FIGURE 7:**
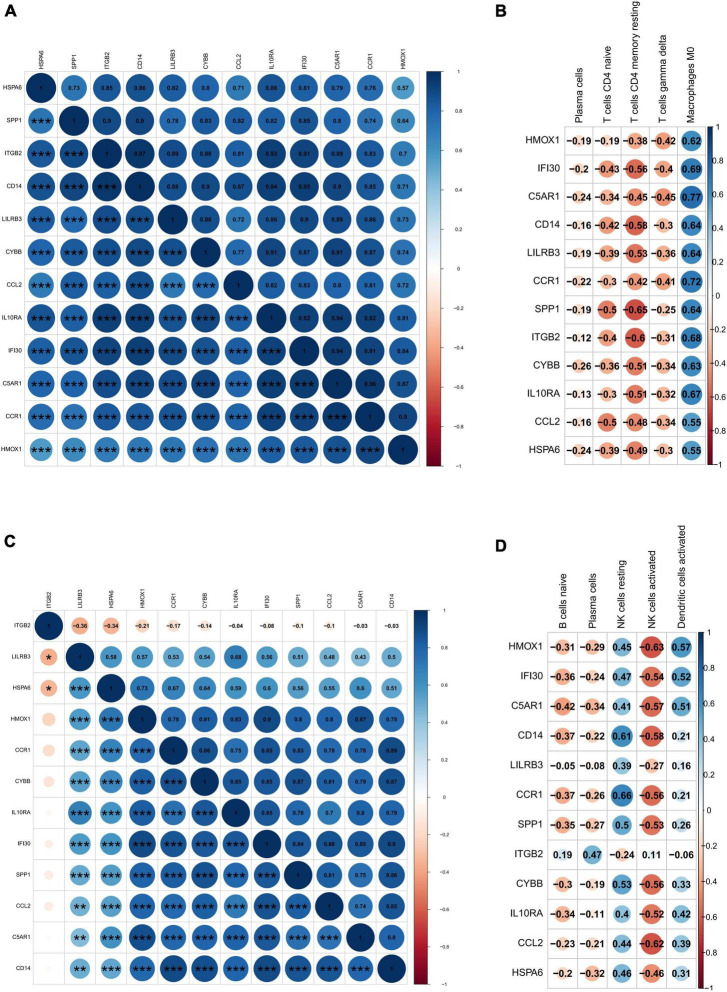
Correlation analysis of differentially expressed immune-related genes and immune cells. **(A)** Heat map of the correlation between differentially expressed immune-related genes in carotid artery plaque samples. **(B)** Heat map of the correlation between differentially expressed immune-related genes in vascular dementia samples. **(C)** Heat map of the correlation between differentially expressed immune-related genes and differentially expressed immune cells in carotid artery plaque samples. **(D)** Heat map of the correlation between differentially expressed immune-related genes and immune cells in vascular dementia samples. Significance level was denoted by *p-value < 0.05, **p-value < 0.01, ***p-value < 0.001.

Twelve DEIRGs were identified by intersecting these overlapping genes with IRGs. We investigated the correlation between these DEIRGs and found tight connectivity of these DEIRGs (Figures 7A,B). In addition, we investigated the correlation between these DEIRGs and differentially expressed immune cells of the corresponding groups. The results revealed that these DEIRGs were positively correlated with M0 macrophages while negatively with gamma delta T cells, resting CD4 memory T cells, plasma cells, and CD4 naive T cells in the carotid plaques group (Figure 7C). In the VaD group, these DEIRGs except the ITGB2 gene were positively correlated with resting NK cells and activated dendritic cells while negatively with naive B cells, activated NK cells, and plasma cells (Figure 7D).

### Receiver operating characteristic analysis

**FIGURE 8:**
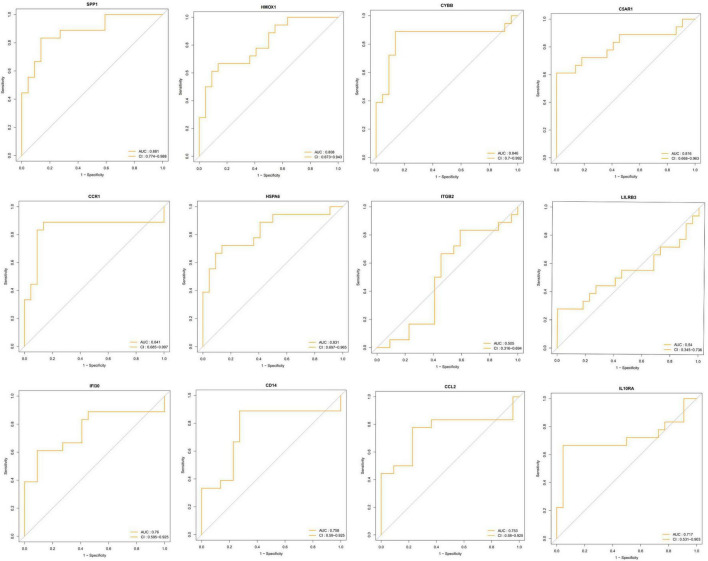
Receiver operating characteristic (ROC) analysis of differentially expressed immune-related genes in the vascular dementia group.

**FIGURE 9:**
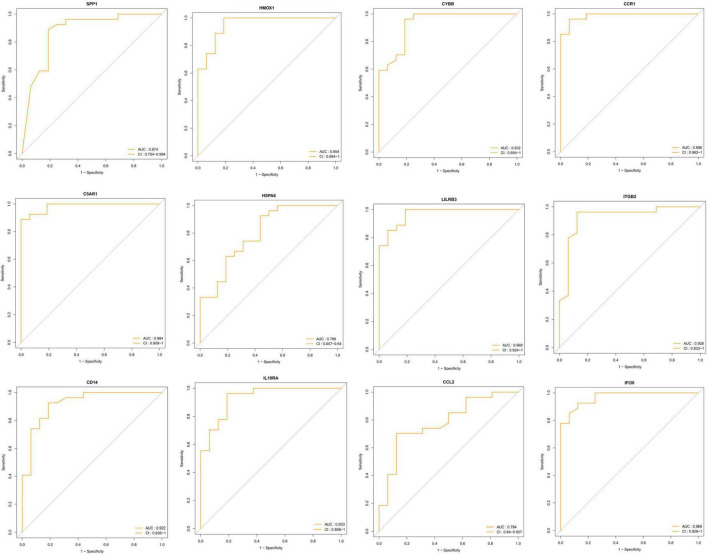
Receiver operating characteristic (ROC) analysis of differentially expressed immune-related genes in the carotid plaque group.

**FIGURE 10:**
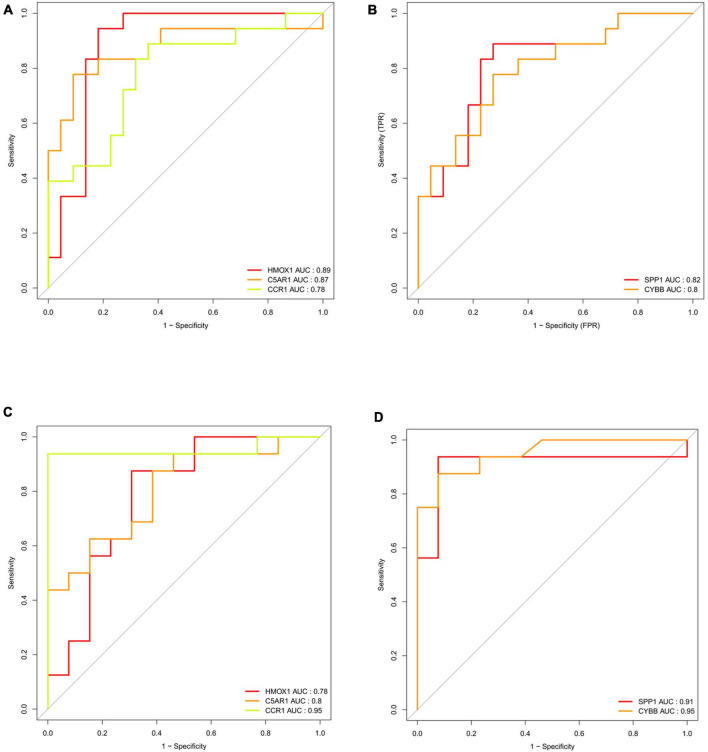
Validation of key genes (HMOX1, C5AR1, CCR1, SPP1, and CYBB) in the validation datasets. **(A)** Receiver operating characteristic (ROC) analysis of HMOX1, C5AR1, and CCR1 in the validation dataset of vascular dementia. **(B)** ROC analysis of SPP1 and CYBB in the validation dataset of vascular dementia. **(C)** ROC analysis of HMOX1, C5AR1, and CCR1 in the validation dataset of carotid plaque. **(D)** ROC analysis of SPP1 and CYBB in the validation dataset of carotid plaque.

To identify the key DEIRGs, we performed the ROC analysis of these DEIRGs (*HMOX1*, *IFI30*, *C5AR1*, *CD14*, *LILRB3*, *CCR1*, *SPP1*, *ITGB2*, *CYBB*, *IL10RA*, *CCL2*, and *HSPA6*) in the VaD (Figure 8) and carotid plaques samples (Figure 9). The results showed that the five genes, including *HMOX1, C5AR1, CCR1, SPP1*, and *CYBB* showed high accuracy in both groups (AUC > 0.80). To further verify the accuracy of these five genes, ROC analysis was performed in the GSE28829 dataset and frontal cortex of the GSE122063 datasets. These five genes showed strong accuracy in identifying vascular dementia (Figures 10A,B) and carotid plaque progression (Figures 10C,D), which indicated the involvement of these five key genes in both carotid plaques and VaD.

### Gene-drug interaction analysis

The five key DEIRGs were considered as potential druggable targets for VaD or carotid plaques treatment. The drug-gene interaction analysis found 19 candidate target drugs/compounds for treatment. Among them, four targeted *HMOX1*, five targeted *CCR1*, five targeted *SPP1*, three targeted *CYBB*, and one targeted *C5AR1*. For 14 of these candidate drugs, no specific interactions with these five key genes have been reported; therefore, these drugs might need further investigation. For the other five candidate drugs, specific types of interactions with key genes have been reported (Table 4). Until now, none of the five drugs have been used directly to treat VaD or carotid plaques, as was shown by searching the ClinicalTrials.gov registry.

**TABLE 4:** Gene-drug interaction analysis.

Gene symbol	Gene name	Drug	Interaction type and directionality	Query score	Interaction score
HMOX1	Heme oxygenase 1	STANNSOPORFIN	N/A	15.06	54.1
HMOX1	Heme oxygenase 2	SUNITINIB	N/A	0.22	0.79
HMOX1	Heme oxygenase 3	ASPIRIN	N/A	0.12	0.43
HMOX1	Heme oxygenase 4	SORAFENIB	N/A	0.11	0.38
C5AR1	Complement C5a receptor 1	AVACOPAN	Antagonist (inhibitory)	4.3	61.83
CCR1	C–C motif chemokine receptor 1	CCX354	Antagonist (inhibitory)	12.91	37.1
CCR1	C–C motif chemokine receptor 2	CHEMBL2205805	N/A	8.61	24.73
CCR1	C–C motif chemokine receptor 3	AZD4818	Antagonist (inhibitory)	4.3	12.37
CCR1	C–C motif chemokine receptor 4	BMS-817399	Antagonist (inhibitory)	4.3	12.37
CCR1	C–C motif chemokine receptor 5	TERPYRIDINE	N/A	1.08	3.09
SPP1	Secreted phosphoprotein 1	ASK-8007	Inhibitor (inhibitory)	4.3	10.3
SPP1	Secreted phosphoprotein 2	CALCITONIN	N/A	1.43	3.43
SPP1	Secreted phosphoprotein 3	ALTEPLASE	N/A	0.45	1.08
SPP1	Secreted phosphoprotein 4	GENTAMICIN	N/A	0.41	0.98
SPP1	Secreted phosphoprotein 5	WORTMANNIN	N/A	0.31	0.74
SPP1	Secreted phosphoprotein 6	TACROLIMUS	N/A	0.25	0.61
CYBB	Cytochrome b-245 beta chain	CHRYSIN	N/A	0.61	2.94
CYBB	Cytochrome b-246 beta chain	APIGENIN	N/A	0.33	1.59
CYBB	Cytochrome b-247 beta chain	LUTEOLIN	N/A	0.27	1.29

Drug-gene Interaction type: In inhibitor interactions, the drug binds to a target and decreases its expression or activity. Most interactions of this class are enzyme inhibitors, which bind an enzyme to reduce enzyme activity (Directionality: inhibitor); An antagonist interaction occurs when a drug blocks or dampens agonist-mediated responses rather than provoking a biological response itself upon binding to a target receptor (Directionality: inhibitory); N/A: DGIdb assigns this label to any drug-gene interaction for which the interaction type is not specified by the reporting source.

## Discussion

In this study, we investigated gene expression patterns from the brain cortex of VaD and atheromatous carotid plaques of symptomatic patients undergoing carotid endarterectomy surgery using a systems biology approach to identify biomolecular signatures that underlie common pathophysiological mechanisms between the two diseases.

We identified 41 overlapped DEGs between the VaD and carotid atherosclerosis plaque datasets. GO enrichment analyses revealed that these overlapped DEGs were mainly enriched in inflammatory and immune-related processes. Previous studies showed that inflammatory and immune-related processes played vital roles in the development of VaD and carotid atherosclerosis plaques (Ammirati et al., 2015; Poh et al., 2021). Our results aligned with previous studies and indicated that inflammatory and immune-related processes might be a crucial common pathophysiological mechanism that VaD and atheromatous carotid plaques shared.

Considering the above functional enrichment analysis results, we further evaluated the infiltration of 22 immune cell types in two datasets. The results revealed that the top five highest infiltrating fractions in carotid plaque and VaD samples differed, indicating that although inflammatory and immune processes were a common pathophysiological mechanism between two diseases, they might be mediated by distinct immune cell types. In carotid atherosclerosis plaque samples, M0 macrophages, M2 macrophages, and T cells gamma delta have a dominant abundance, consistent with a previous study that showed that T cells and macrophages dominated the immune landscape of atherosclerotic plaques (Fernandez et al., 2019). Moreover, M0 macrophages showed a significantly different infiltration percentage between the early and advanced stage plaques group. Plaque macrophages are a functionally heterogeneous group of immune cells, and M0 macrophages were considered non-differentiated macrophages, which could polarize toward pro-inflammatory M1 macrophages or anti-inflammatory M2 macrophages. M1 macrophages could contribute to an increased and sustained inflammatory response via the secretion of pro-inflammatory cytokines and promote the progression of atherosclerotic plaques. In contrast, the M2 macrophages are considered anti-atherogenic by producing anti-inflammatory cytokines and promoting tissue repair (Colin et al., 2014). Therefore, it is necessary to elucidate the molecular mechanisms of macrophage phenotype switching in the future, which might be a potential target for delaying plaque progression.

**FIGURE 11:**
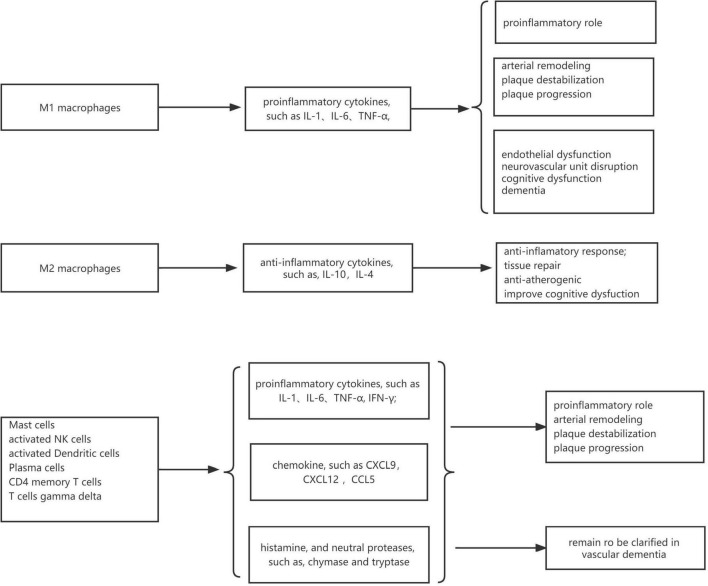
The association between inflammation pathways and the immune system as well as the development of vascular dementia and carotid plaque.

In VaD samples, resting CD4 memory T cells, M2 macrophages, and naive B cells were the top three highest infiltrating fractions. Furthermore, B cells and NK cells showed a different infiltration percentage between VaD and matched controls. Previous studies have rarely focused on the infiltration of immune cell types in VaD. Busse et al. (2017, 2021) investigated the frequency of innate and adaptive immune cell populations in whole blood and CSF from different forms of dementia, including VaD. They found alterations of innate and adaptive immune cells in the CSF and peripheral immune system. Compared to healthy persons, B cells, T cells, monocytes, and NK cells in whole blood were diminished in VaD, and central memory CD4 + T cells in CSF were reduced, and NK and NKT cells were enhanced in VaD; the number of B lymphocytes was unchanged between the VaD and controls. Together, these results indicated that these immune cell types might be involved in the pathogenesis of VaD, and their role needs to clarify in the future. We identified 12 DEIRGs and investigated the correlation between these DEIRGs and differentially expressed immune cells of the corresponding groups. The results revealed that these DEIRGs were closely related to differentially expressed immune cells, indicating that these DEIRGs might have an essential role in altering the infiltration percentage of these immune cells. Figure 11 briefly summarizes the association between inflammation pathways and the immune system as well as the development of vascular dementia and carotid plaque.

We identified five key DEIRGs based on ROC analysis. The gene *HMOX1* also named *HO-1*, encodes the enzyme that could exert its anti-oxidant and anti-inflammatory effects by several pathways, including degrading the pro-inflammatory heme and producing potent anti-oxidants (Nath et al., 2000). Moreover, as one of the Nrf2 targets, HO-1 had neuroprotective effects. HO-1 knockout worsens infarcts (Shah et al., 2011), and HO-1 overexpression reduces infarcts in mice (Zhang et al., 2012). Mao et al. (2019) induced experimental vascular cognitive impairment in rats by permanent occlusion of both common carotid arteries for 6 weeks. They found sulforaphane could alleviate neuronal damage, white matter injury, blood-brain barrier disruption cognitive impairment by activating the Nrf2/HO-1 pathway. HO-1 also played a protective role in the pathophysiology of atherosclerosis. In APOE-/- mice, a lack of HO-1 accelerated atherosclerosis (Yet et al., 2003), whereas HO-1 induction inhibited atherosclerotic lesion formation in low-density lipoprotein receptor knockout mice (Ishikawa et al., 2001). A study found that plasma HO-1 levels were significantly higher in subjects with carotid plaque than without plaque and were a significant factor for carotid plaque independent of atherosclerotic risk factors (Kishimoto et al., 2018). The protein CD88 encoded by the gene *C5AR1* is a classical C5a receptor that has a role in CNS disease processes by binding to C5a. A previous study showed increased expression of CD88 in VaD brain tissue compared to age-matched controls or AD samples by Western blot and immunohistochemistry, which was consistent with our results (Fonseca et al., 2013). Another study showed that treatment with a CD88-specific antagonist enhanced behavioral performance in murine AD models, suggesting that C5a/CD88 interaction had an essential role in the progression of AD (Fonseca et al., 2009). However, the role of CD88 in VaD was not clear. Pavlovski et al. (2012) found that the C5a and CD88 were up-regulated in ischemic neurons and promoted neuronal apoptosis. Pretreatment of the cells with the specific CD88 receptor antagonist PMX53 significantly blocked ischemia-induced apoptosis. They also found that CD88–/– mice subjected to middle cerebral artery occlusion (MCAO) had significantly reduced infarct volumes and improved neurological scores. Stroke is one of the leading causes of VaD; thus, reducing ischemic injury might help prevent or delay the progression of VaD. Accumulating evidence in humans and experimental animal models revealed that the C5a–C5aR axis is involved in the development of atherosclerosis lesions (An et al., 2014). A recent study found that C5aR1 was higher in carotid plaques than in control arteries (Niyonzima et al., 2020), which was in line with our results. The gene *CCR1* encodes a member of beta chemokine receptor family that belongs to G protein-coupled receptors (White et al., 2013). The activation of CCR1 was pro-inflammatory in several animal models of neurological diseases. A previous study reported that the expression of CCR1 was increased in the brain after MCAO (Offner et al., 2006) and intracerebral hemorrhage (ICH) in mice (Yan et al., 2020). In the mouse model of ICH, CCR1 activation could promote acute neuroinflammation, and the inhibition of CCR1 improved neurobehavioral deficits and attenuated brain edema and neuroinflammation (Yan et al., 2020). These results indicated that CCR1 might be involved in the pathophysiological mechanism of stroke. There are few studies on the role of CCR1 in atherosclerosis. A study found that blood-borne CCR1 altered the immuno-inflammatory response in atherosclerosis and prevented excessive plaque growth and inflammation (Potteaux et al., 2005). A recent study also found that CCR1 was up-regulated in carotid atherosclerosis, but the exact mechanism was unclear (Meng et al., 2021). The gene *SPP1* encodes the protein secreted phosphoprotein 1, also known as osteopontin (OPN). OPN is an extracellular phosphoprotein expressed in various tissues and cells in response to stress and injury and is also a soluble cytokine involved in inflammation. Previous studies revealed that it was upregulated in symptomatic carotid atherosclerosis (Golledge et al., 2004), stroke (Carbone et al., 2015), and vascular cognitive impairment (Chai et al., 2021), suggesting the involvement of OPN in these inflammation-associated neurological diseases, which supported our results. The gene *CYBB* encodes the protein cytochrome b-245 heavy chain, also named NADPH oxidase 2 (Nox 2), which is one of the NADPH oxidase (Nox) isoforms. It was reported that Nox 2 had been implicated in the VaD and atherosclerotic plaques. Previous studies indicated that Nox2 hereditary deficiency in humans was associated with reduced atherosclerotic burden (Violi et al., 2013). Specific Nox2 inactivation would arrest atheroma plaque progression and instability in animal models (Quesada et al., 2015). Another study revealed that Ling-Yang-Gou-Teng -Decoction, a well-known traditional Chinese formula, exhibited beneficial effects on the VaD by decreasing NOX2 expression (Zhao et al., 2018). Collectively, these results indicated that the five key genes played vital roles in the pathogenesis of VaD and carotid atherosclerotic plaques and might be potential targets for both diseases’ treatment.

We performed a drug-gene interaction analysis to detect potential target drugs/compounds for VaD and carotid atherosclerotic plaques treatment and identified five drugs that were predicted to have specific types of interactions with these key genes. We checked the five candidate drugs in the ClinicalTrials.gov registry. Four drugs (avacopan, CCX354, BMS-817399, and ASK-8007) could be potential drugs for VaD and carotid atherosclerotic plaques treatment. Avacopan was a C5AR1 antagonist, CCX354 and BMS-817399 were CCR1 antagonists, and ASK-8007 was an SPP1 inhibitor. A randomized, placebo-controlled trial showed that C5a receptor inhibition with avacopan was effective in replacing high-dose glucocorticoids in treating antineutrophil cytoplasmic antibody (ANCA)-associated vasculitis. CCX354-C, BMS-817399, and ASK-8007 were all studied to treat Rheumatoid arthritis (RA). ANCA-associated vasculitis and RA were both chronic inflammatory diseases and associated with atherosclerosis and VaD (Atzeni et al., 2017). A systematic review with meta-analysis showed a statistically significant increase in the risk of dementia among patients with RA compared with non-RA controls (Ungprasert et al., 2016). Bilateral carotid plaque was more than twice in RA than in controls (Wah-Suarez et al., 2019). In future studies, these four drugs need to be evaluated as potential drugs in VaD and carotid atherosclerotic plaques treatment.

There are some limitations to the present study. Firstly, the study is only based on bioinformatics analysis; no clinical or experimental confirmation of the roles of key genes was attempted. Secondly, the sample size in this study is not very large, and it will be necessary to replicate our results in more datasets with larger sample size when available. Notwithstanding these limitations, this study offers some insights into the underlying common pathophysiological mechanism shared by VaD and carotid plaques and might provide potential targets for the treatment.

## Conclusion

We identified overlapping DEGs between VaD and carotid plaques. These overlapping DEGs were enriched mainly in inflammatory and immune-related processes, indicating it might be a crucial common pathophysiological mechanism shares by VaD and carotid plaques. We identified DEIRGs, evaluated the infiltration of immune cells, and investigated the correlation between DEIRGs and differentially expressed immune cells to gain greater insight into the role of inflammatory and immune-related processes in vascular dementia and carotid plaque progression. We identified five key DEIRGs and performed gene-drug analysis, which might provide potential targets and drugs for therapeutic intervention.

## Data availability statement

The original contributions presented in this study are included in the article/supplementary material, further inquiries can be directed to the corresponding authors.

## Author contributions

JS, YR, WT, WW, LZ, and JC conceived and designed the study, analyzed the data, wrote the manuscript, contributed to the designs of the methods used in this study, read, and agreed to the publication of this version of the manuscript.

## Footnotes

1
https://www.ncbi.nlm.nih.gov/geo/

2
https://www.ncbi.nlm.nih.gov/geo/geo2r/

3
http://metascape.org

4
www.dgidb.org
